# Association of *FLG* single nucleotide variations with clinical phenotypes of atopic dermatitis

**DOI:** 10.1371/journal.pone.0190077

**Published:** 2017-12-27

**Authors:** Myungshin Kim, Jaeeun Yoo, Jiyeon Kim, Joonhong Park, Eunhee Han, Woori Jang, Hyojin Chae, Ji Hyun Lee, Young Min Park, Yonggoo Kim

**Affiliations:** 1 Department of Laboratory Medicine, College of Medicine, The Catholic University of Korea, Seoul, Republic of Korea; 2 Catholic Genetic Laboratory Center, Seoul St. Mary’s Hospital, College of Medicine, The Catholic University of Korea, Seoul, Republic of Korea; 3 Center for Applied Genomics and Precision Medicine, Seoul St. Mary’s Hospital, College of Medicine, The Catholic University of Korea, Seoul, Republic of Korea; 4 Department of Dermatology, College of Medicine, The Catholic University of Korea, Seoul, Republic of Korea; INSERM, FRANCE

## Abstract

**Background:**

*FLG* encodes a large protein called profilaggrin, which plays a key role in maintaining an effective skin barrier against the environment. In this study, we identified *FLG* single nucleotide variations (*FLG*-SNVs) and evaluated the association of *FLG*-SNVs with clinical phenotypes including atopic dermatitis (AD)-associated minor clinical features, presence of specific allergic sensitization, and serum parameters.

**Methods:**

Eighty-one Korean patients with AD were enrolled. AD-associated minor clinical features as well as allergic rhinitis and asthma were diagnosed by specialists. *FLG*-SNVs were identified by Sanger sequencing of entire exons through long-range PCR. Allergic sensitization to a specific allergen was evaluated by multiple allergen simultaneous test. Serologic parameters such as serum eosinophil cationic protein (ECP) and eosinophil derived neurotoxin (EDN) were measured.

**Results:**

A total of seventy-three SNVs and 4 LOF mutations were successfully genotyped. rs71626704 and rs76413899 were significantly associated with a history of asthma and cheilitis (*P* = 0.002 and *P* = 0.033, respectively), however, the associations were not found statistically significant after adjustment by multiple comparisons. In addition, we detected haplotype blocks which were correlated with non-specific hand or foot dermatitis and scalp scale. We identified *FLG*-SNVs which were associated with sensitization to environmental allergens; rs62623409 and rs71625199 (*P* = 0.038 and *P* = 0.008, respectively). Patients with *FLG* P478S TT and history of allergic rhinitis showed a higher EDN level, and among those patients, the ones with asthma showed a higher ECP level.

**Conclusion:**

This study revealed the association of *FLG*-SNVs with AD-associated minor clinical features. We firstly identified rs71625199 which was associated with higher environmental allergic sensitization. We also suggest that *FLG* P478S is a kind of disease modifier which affects serologic parameters such as EDN and ECP.

## Introduction

Atopic dermatitis (AD) is a pruritic, inflammatory skin disease with a chronic relapsing course. This disease has been known to be influenced by environmental and genetic factors [1]. Profilaggrin is cleaved to produce multiple copies of the filaggrin protein, which plays a key role in maintaining an effective skin barrier against the environment [2–5]. The filaggrin gene loss-of-function (*FLG*-LOF) mutations lead to reduce protein expression, making the skin more permeable to environmental allergens and increasing trans-epidermal water loss [6–8]. *FLG*-LOF mutations have been identified as a risk factor for allergic sensitization, and allergic rhinitis and asthma [9–11]. Not only *FLG*-LOF mutations but also mutations associated with epidermal barriers and immune response have also been linked to AD [12, 13]. In addition, one *FLG* single nucleotide variation (*FLG*-SNV) corresponding to P478S has been reported to have a clinical implication in AD patients [14] and to increase the risk of asthma combined with allergic sensitization [15]. *FLG*-LOF has been recognized as a disease-modifying factor in AD strongly associated with early onset and severe disease in European populations [8].

In this study, we analyzed *FLG*-SNVs by Sanger sequencing of entire exons through long-range PCR and evaluated the association of *FLG*-SNVs with clinical phenotypes such as AD-associated minor clinical features, presence of specific allergic sensitization, and serum parameters in AD patients.

## Materials and methods

### Patients

Eighty-one Korean patients with AD were recruited. The mean age of the study group was 15.25 ± 8.07 years and 56.8% of patients were male. Family history of AD was found in 53.1% of patients and 18.5% of patients had a history of early onset disease. These patients were outpatients referred to the Department of Dermatology, Seoul St Mary’s Hospital. Detailed phenotypic data, especially AD-associated minor clinical features, including xerosis, pityriasis alba, cheilitis, tendency towards non-specific hand or foot dermatitis (NS-HFD), scalp scale, perifollicular accentuation, keratosis pilaris, palmar hyperlinearity, and ichthyosis, were gathered and examined by experienced dermatologists. Allergic rhinitis and asthma were diagnosed based on the questionnaire answers and the diagnosis made by pediatricians or otolaryngologists. Subjects were informed about the study design and purpose according to the Declaration of Helsinki. All subjects of the study provided informed consent, and the protocol of the study was approved by the institutional review board (KC10TISI0582) of The Catholic University of Korea.

### Genotype analysis of the *FLG* gene

Genomic DNA samples were extracted from the patients’ peripheral blood using the QIAamp DNA Mini Kit (Qiagen, Hilden, Germany). DNA from one hundred healthy, unrelated individuals was used as the non-selected population control. Coding exons of *FLG* were amplified by polymerase chain reaction (PCR) using an overlapping long distance PCR strategy (Expand Long Template PCR System; Roche, Basel, Switzerland) described elsewhere [16]. After PCR, bidirectional sequencing was performed with the BigDye Terminator v3.1 Cycle Sequencing Kit (Applied Biosystems, Foster City, CA, USA). The products were resolved on an ABI 3130XL Genetic Analyzer (Applied Biosystems). Sequence electropherogram was analyzed by the Sequencher Software 4.9 (Gene Codes, Ann Arbor, MI, USA). Sequence data were analyzed using reference sequences in GenBank. cDNA nucleotide numbering was performed using *FLG* Reference sequence ID NM_002016.1. *FLG* variants were analyzed using the Human Gene Mutation Database (HGMD, http://www.hgmd.cf.ac.uk/), ClinVar (http://www.ncbi.nlm.nih.gov/clinvar/), the 1000 Genomes Project (http://browser.1000genomes.org/), the Exome Aggregation Consortium (ExAC, http://exac.broadinstitute.org/) and the Korean Reference Genome Database (KRGDB, http://152.99.75.168/KRGDB/menuPages/intro.jsp), which contains whole genome sequencing data for 622 Korean individuals [17–19].

### Computational prediction tools

Several prediction tools were used to evaluate the *FLG* mutations identified in Korean AD patients. The following widely established computational prediction methods were applied: the evolution-based prediction tools including Sorting Intolerant From Tolerant (SIFT, http://sift.jcvi.org/), and protein structure and function were assessed by using straightforward comparative physical and evolutionary analyses and sequence- and structure-based bioinformatics tools, such as Polymorphism Phenotyping v2 (PolyPhen-2, http://genetics.bwh.harvard.edu/pph2/) [19, 20].

### Eosinophil cationic protein and eosinophil derived neurotoxin

Serum eosinophil cationic protein (ECP) and eosinophil derived neurotoxin (EDN) levels were measured. Both were secreted by activated eosinophils; therefore, they were considered as sensitive markers for disease activity in AD [21, 22]. We measured the ECP level using a solid-phase, 2-site chemiluminescent immunometric assay on an Immulite 2000 analyzer (Siemens Healthcare Diagnostics, Tarrytown, N.Y., USA). The cutoff limit for a positive serum ECP result was set at >19 μg/L on the basis of the manufacturer's instructions. The EDN level was analyzed using The BioTracer™ K® EDN ELISA Kit according to the manufacturer’s instructions. After reaction, absorbance was measured at 450 nm by a Micro Plate Reader Infinite 200 PRO (TECAN, Mannedorf, Switzerland). The concentration obtained from the standard curve after calculating the average absorbance values for each set of duplicate standards and duplicate samples. The analytical measurement range of the EDN assay is 6.0 to 400 ng/mL. AD patients included in this study showed significantly higher levels of both ECP and EDN compared to healthy controls (59.12 μg/L vs. 17.04 μg/L, *P*<0.001, 110.51 ng/mL vs. 43.44 ng/mL, *P*<0.001, respectively).

### Specific allergic sensitization

Multiple allergen simultaneous test (MAST) was performed to analyze the serum-specific IgE using Advansure Alloscreen (AS: LG Life Science, Seoul, Korea). A total of 20 allergens commonly present in AD patients were included; two mite allergens (*Dermatophagoides farinae* (Df), *Dermatophagoides pteronyssinus* (Dp)), 8 food allergens (egg, milk, soy bean, wheat flour, shrimp, chicken, peanut, and pork), and 10 environmental allergens (cat epithelium, dog dander, cockroach, birch-alder mix, oak white, ragweed short, mugwort, *Alternaria alternata*, grass mix, and Japanese hop). Briefly, 250 μL of serum was reacted to the allergen on a test strip. After washing, antihuman IgE antibody coupled with biotin was added and incubated. Then, unbound antibody was removed, which was followed by incubation with streptavidin conjugated to alkaline phosphatase. After incubation with luminescent solution, test strips were completely dried and read using AdvanSure AlloScan. Specific IgE concentration was classified from 0 to 6, and results greater than the levels in class 2 (sIgE ≥ 0.7 kU/L) were considered positive.

### Statistical analysis

Chi-square test was used to compare the allele frequencies of *FLG*-SNVs according to AD-associated minor clinical features. Odds ratios (OR) and 95% confidence intervals (95% CI) were calculated from logistic regression analysis. Haploview was used to generate a linkage disequilibrium (LD) map by using Gabriel’s rule. Tests for associations using multi-marker haplotypes were performed using the statistics software ‘‘R” (http://www.R-project.org), package ‘haplo.stat’. To assess the population impact of the variants we identified, we performed a case-control analysis of each *FLG*-SNVs. The genotype and allele frequencies for patients and control subjects were used to calculate the odds ratios (ORs). We used the allele data of East Asian population in Exome Aggregation Consortium for the control group. Multiple comparisons were performed by one-way ANOVA and Bonferroni correction. A *P* value of less than 0.05 was considered statistically significant. PLINK software program (http://pngu.mgh.harvard.edu/~purcell/plink/), Haploview program (http://www.broad.mit.edu/mpg/haploview), and SAS program (version 9.2; SAS Institute Inc., Cary, NC, USA) were used for statistical analysis.

## Results

### *FLG*-SNV and AD-associated minor clinical features

A total of seventy-three SNVs and 4 LOF mutations were successfully genotyped in this study. The data set are provided in Supplementary table (S1 Table). The results comparing their frequencies to normal East Asian population and *in silico* prediction are listed in S2 Table. We analyzed the interaction of SNVs with AD-associated minor clinical features including NS-HFD, asthma, cheilitis, and scalp scale. The A allele frequency of rs71626704 was significantly higher in patients with asthma (37.5%, 3/8) than in patients without asthma (4.8%, 3/62) (*P* = 0.002). The allele A of rs76413899 was only identified in patients with cheilitis (16.3%, 7/43) (*P* = 0.033). NS-HFD was associated with *FLG*-SNVs including P478S (S3 Table). A Bonferroni correction was applied for multiple comparisons with all *FLG*-SNV associations, with a correction factor derived from the number of SNVs tested (for 73 comparisons, critical *P* value is 0.000685). However, no significant associations were found between any single *FLG*-SNVs and AD-associated minor clinical features.

**Fig 1:**
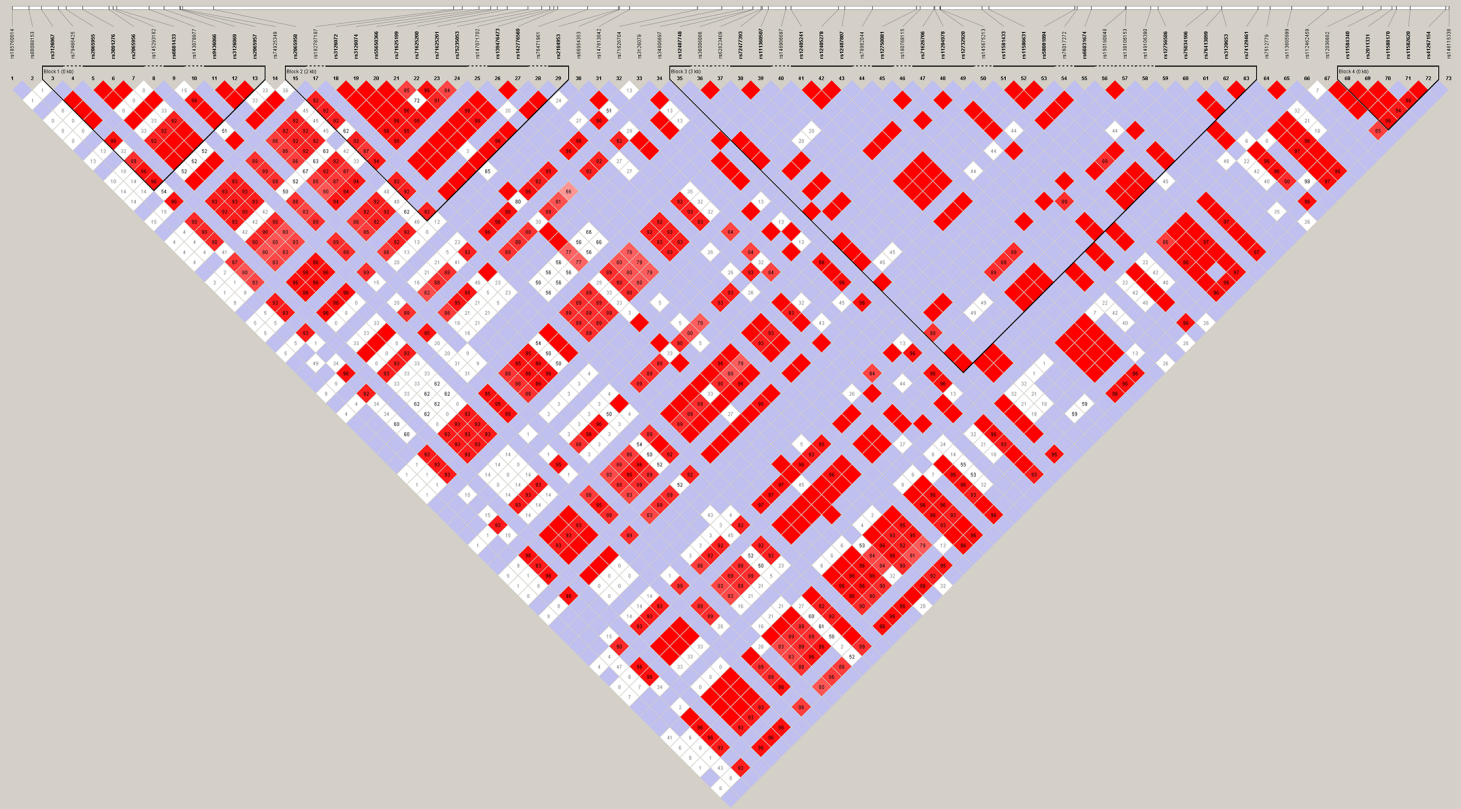
Linkage disequilibrium map of the *FLG* gene in atopic dermatitis patients.

In addition, we detected four haplotype blocks after linkage analysis (Fig 1) (Block 1, rs3126067, rs2065955, rs3091276, rs2065956, rs6681433, rs9436066, rs3126069, rs2065957; Block 2, rs2184953, rs139476473, rs142776569, rs3126072, rs3126074, rs55650366, rs71625199, rs71625200, rs71625201, rs75235053, rs2065958; Block 3, rs12756586, rs75034106, rs76413899, rs3120653, rs74129461, rs66831674, rs11581433, rs11586631, rs58001094, rs71626706, rs11204978; Block 4, rs11584340, rs2011331, rs11588170, rs11582620, rs41267154) and their correlation with AD-associated minor clinical features including NS-HFD and scalp scale (Table 1).

**Table 1:** Haplotype blocks of the *FLG* gene related to atopic dermatitis-associated minor clinical features.

AD-associated feature	Block	Haplotypes	Control frequency	Case frequency	*P* value
NS-HFD	Block 2	CATAGCGGCCG	0.36	0.14	0.0139
		TGCGCTGAGGG	0.03	0.15	0.0173
	Block 3	CCGCACGAGCATCTTATGA	0.33	0.03	0.0079
		CTGTGTAGCCCTCCCGTGG	0.12	0.42	0.0259
Scalp scale	Block 2	TGCGCTGAGGT	0.46	0.29	0.016
		CATAGCGGCGG	0.02	0.11	0.0384
	Block 3	CTGTGTAGCCCTCCCGTGG	0.15	0.24	0.0068
		CCGCACGAGCATCTTATGA	0.24	0.19	0.0187
	Block 4	TGATT	0.18	0.08	0.0052

AD, atopic dermatitis; NS-HFD, nonspecific-hand or foot dermatitis; Block 2, rs2184953-rs139476473-rs142776569-rs3126072-rs3126074-rs55650366-rs71625199-rs71625200-rs71625201-rs75235053-rs2065958; Block 3, rs12756586-rs75034106-rs76413899-rs3120653-rs74129461-rs66831674-rs11581433-rs11586631-rs58001094-rs71626706-rs11204978; Block4, rs11584340-rs2011331-rs11588170-rs11582620-rs41267154.

### *FLG*-SNV and specific allergic sensitization

MAST determined specific allergic sensitization in 63 patients (77.8%). Mite allergens including Df and Dp were the most commonly sensitized allergens (56.1% and 58.5%, respectively). Patients with mite allergic sensitization showed a higher risk of allergic rhinitis (*P* = 0.025, OR 3.018, 95% CI 1.136–8.016). Although we did not identify the association of *FLG*-SNV with sensitization to mite and food allergens, we found that *FLG*-SNVs were associated with sensitization to other environmental allergens. Among the six patients with rs62623409 (Q1873K), five showed environmental allergic sensitization (*P* = 0.038). Patients with allele A of rs71625199 showed a higher risk of sensitization to environmental allergens (*P* = 0.008, OR 4.173, 95% CI 1.454–11.972). We found eight *FLG*-SNVs which were significantly associated with dog dander allergic sensitization (Table 2). Interestingly, patients with any of these SNVs showed a higher risk of sensitization to dog dander allergen compared to patients without these eight *FLG*-SNVs (*P* = 0.002, OR 9.091. 95% CI 1.893–43.657). In our study, we did not find any association between *FLG*-LOF mutations and the presence of specific allergic sensitization.

**Table 2:** Association of *FLG* single nucleotide variation (SNV) with dog dander allergic sensitization.

*FLG*-SNV	Dog dander allergic sensitizaion		*P* value
	Positive(%)	Negative(%)	
rs7512779	3.2	25.0	0.003
rs12756586	4.8	25.0	0.011
rs76017272	0.0	12.5	0.004
rs12732920	4.8	25.0	0.011
rs71626706	4.8	25.0	0.011
rs12750081	4.8	25.0	0.011
rs62623409	3.2	25.0	0.003
rs71626704	3.2	25.0	0.003

### *FLG*-SNV and serum parameters

**Fig 2:**
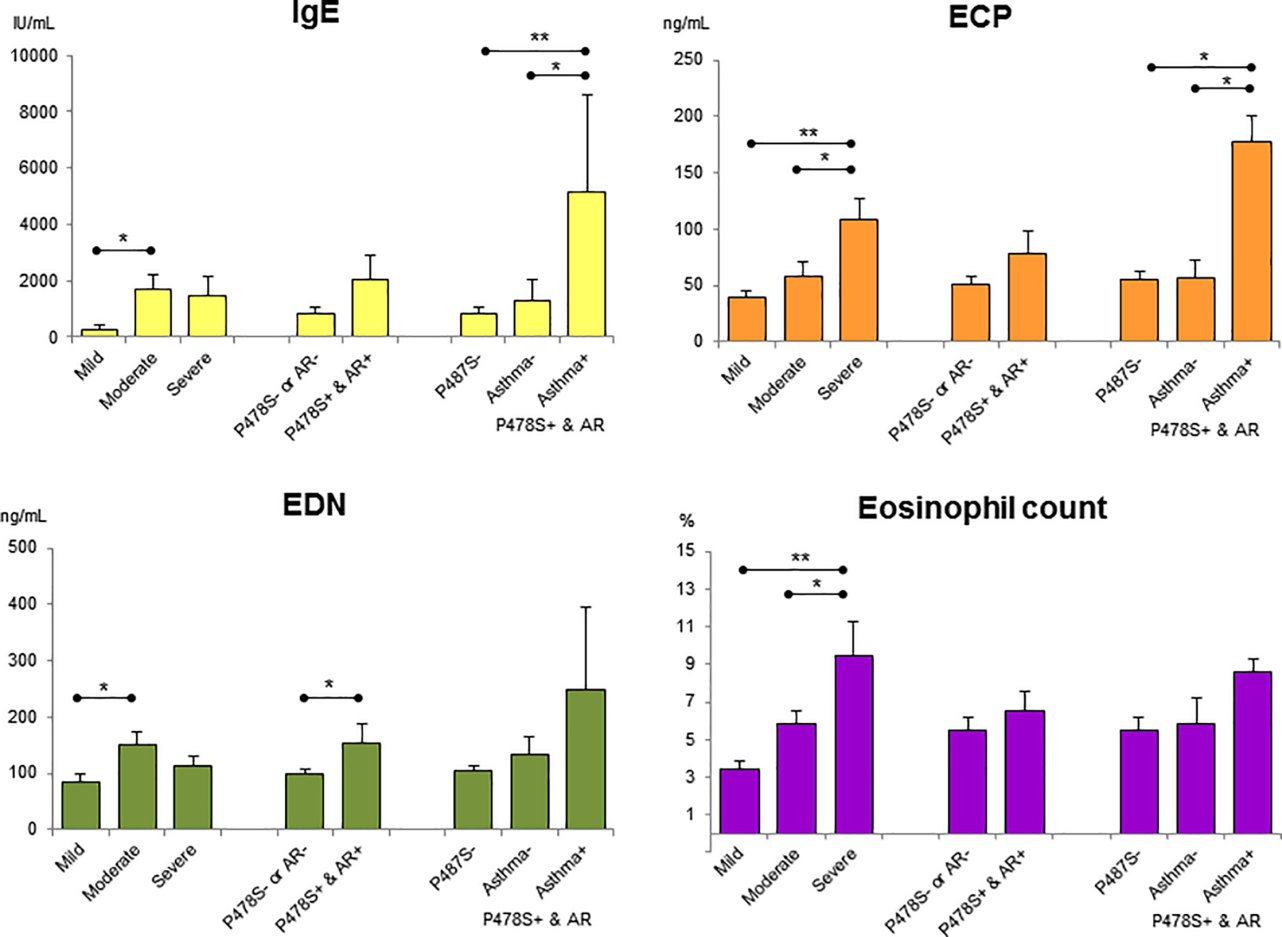
Comparison of serum parameters in atopic dermatitis according to scoring atopic dermatitis (SCORAD), presence or absence of *FLG* P478S, and history of allergic rhinitis (AR) and asthma among patients with P478S and AR. * *P* <0.05, ** *P* <0.01.

Scoring atopic dermatitis (SCORAD) is a clinical tool used to assess the extent and severity of eczema. Dermatologists may use this tool before and after treatment to determine whether the treatment has been effective [23]. In this study, serum IgE, ECP, and EDN levels, and eosinophil count were significantly different according to SCORAD (*P* = 0.028, *P* = 0.001, *P* = 0.022 and *P* < 0.001, respectively). EDN level was higher in the moderate group compared to the mild group (*P* = 0.018), while ECP level was higher in the severe group compared to the mild and moderate group (*P* = 0.001 and *P* = 0.028, respectively). Eosinophil count was higher in severe group compared to the mild and moderate group (*P* < 0.001 and *P* = 0.037) (Fig 2). Patients with *FLG* P478S TT and history of allergic rhinitis (n = 11) showed a higher EDN level (154.5 ± 34.7 ng/mL vs. 99.1 ± 9.3 ng/mL, *P* = 0.041). Serum IgE and ECP levels were significantly different according to *FLG*-SNV and history of allergic rhinitis and asthma (*P* = 0.009 and *P* = 0.013, respectively). Patients with *FLG* P478S TT and history of allergic rhinitis and asthma showed a higher IgE and ECP level compared to those without asthma (*P* = 0.037 and *P* = 0.022) and patients without P478S TT (*P* = 0.008 and *P* = 0.010).

## Discussion

AD is a complex disease and genetic and environmental factors are implicated in the heterogeneity of AD. Because filaggrin is a multifunctional protein that can alter the skin barrier function and inflammatory response [24], *FLG*-SNVs as well as *FLG*-LOF may have an influence on the clinical phenotype of AD. Here, we performed *FLG* sequencing including all exons and flanking introns and identified most of the known SNVs (n = 73). We searched public databases of genetic variants including the KRGDB and performed *in silico* analyses of the identified missense variants. To the best of our knowledge, this is the first study to investigate the correlation of *FLG*-SNVs with clinical phenotypes including AD-associated minor clinical features, specific allergic sensitization, and serum parameters. rs71626704 (D2015E), rs76413899 (G837S), and rs11584340 (P478S) were significantly associated with the development of asthma, cheilitis, and NS-HFD, respectively. The linkage analysis showed a few meaningful haplotype blocks associated with NS-HFD and scalp scale.

The reduction or deterioration of filaggrin influences the skin barrier formation and makes the patient’s skin susceptible to environmental allergens [25] because allergic sensitization occurs through the damaged skin barrier [26]. A previous study found associations between the *FLG*-LOF mutations and sensitizations to grass, house dust mite and cat dander [27]. Few studies have demonstrated that *FLG*-SNV was associated with allergic sensitization in AD patients. We identified *FLG*-SNVs including rs62623409 (Q1873K) which were associated with sensitization to environmental allergens. In addition, rs71625199 was a significant *FLG*-SNV associated with sensitization to environmental allergens. SIFT and polyphen-2 predicted rs62623409 as tolerate and benign, respectively. Even rs71625199 does not induce amino acid change (synonymous). Another *in silico* prediction tool predicted that rs71625199 induce the alteration of splicing through the activation of an exonic cryptic acceptor site [28, 29]. Filaggrin is highly polymorphic and filaggrin itself breaks down, the resulting products contribute to the formation of natural moisturizing factor, which is important for epidermal hydration and barrier function [30]. Therefore, the functional change of the *FLG*-SNV cannot be directly expected via *in silico* analysis.

Currently, there is no reliable serum parameter that can distinguish AD from other disease entities [31]. Although the parameter has little diagnostic significance, several characteristic laboratory changes occur in AD patients. Eosinophils play a role in the inflammatory process of AD by which the activated eosinophils release granule proteins such as ECP and EDN. ECP is a cytotoxic agent secreted by activated eosinophils during allergic and inflammatory processes and it is elevated in AD patients [32]. EDN may also be useful to determine eosinophil activity in allergic diseases [22]. This study demonstrated that serum parameters including ECP and EDN were significantly different according to SCORAD. EDN level was higher in the moderate and severe group compared to the mild group, while ECP level was higher in the severe group compared to the mild and moderate group. Patients with *FLG* P478S TT and history of allergic rhinitis showed a higher EDN level, and among them, patients with asthma showed a higher ECP level. These results strengthen the findings of the previous study which demonstrated that EDN reflects strongly disease severity in AD [33] and increased ECP levels correspond to asthma symptom onset [34]. *FLG* P478S was suggested to hamper protease cleavage and it was associated with the risk of developing asthma or allergic rhinitis [15, 35]. We consider that P478S may act as a modifier of AD especially associated with allergic rhinitis and asthma represented by serologic parameters suggesting disease severity.

There are acknowledged limitations of this study. First, although we found a statistically significant association between *FLG*-SNVs and clinical phenotypes, it was not individually supported by multiple comparisons, previous investigation or accepted physiologic models. Therefore, it remained to be supplemented with an experimental evidence of the functional role of the SNVs. In addition, the pathomechanisms of filaggrin or filaggrin-modifying natural moisturizing factors in atopic dermatitis are complex and more works are required to establish influences *FLG*-SNVs on epidermal barrier defects [36].

In conclusion, this study revealed the association of *FLG*-SNVs with AD-associated minor clinical features such as asthma, cheilitis, NS-HFD, and scalp scale. There is a possibility that *FLG*-SNV rs71625199 may be associated with higher environmental allergic sensitization. We also suggest that *FLG* P478S is a kind of disease modifier which affects serologic parameters such as EDN and ECP.

## Supporting information

S1 Table *FLG* genotypes and clinical information of 81 AD patients. (XLSX)

S2 Table Characteristics of *FLG* single nucleotide variations identified in atopic dermatitis. (XLSX)

S3 Table *FLG* single nucleotide variations associated with non-specific hand or foot dermatitis. (XLSX)

## References

[1] Bisgaard H, Simpson A, Palmer CNA, Bønnelykke K, McLean I, Mukhopadhyay S, et al Gene-Environment Interaction in the Onset of Eczema in Infancy: Filaggrin Loss-of-Function Mutations Enhanced by Neonatal Cat Exposure. PLOS Medicine. 2008;5(6):e131 doi: 10.1371/journal.pmed.0050131 18578563 10.1371/journal.pmed.0050131 PMC2504043

[2] Baurecht H, Irvine AD, Novak N, Illig T, Bühler B, Ring J, et al Toward a major risk factor for atopic eczema: meta-analysis of filaggrin polymorphism data. J Allergy Clin Immunol. 2007;120(6):1406–12. doi: 10.1016/j.jaci.2007.08.067 17980411 10.1016/j.jaci.2007.08.067

[3] Cork MJ, Danby SG, Vasilopoulos Y, Hadgraft J, Lane ME, Moustafa M, et al Epidermal barrier dysfunction in atopic dermatitis. J Invest Dermatol. 2009;129(8):1892–908. doi: 10.1038/jid.2009.133 19494826 10.1038/jid.2009.133

[4] Palmer CNA, Irvine AD, Terron-Kwiatkowski A, Zhao Y, Liao H, Lee SP, et al Common loss-of-function variants of the epidermal barrier protein filaggrin are a major predisposing factor for atopic dermatitis. Nat Genet. 2006;38(4):441–6. doi: 10.1038/ng1767 16550169 10.1038/ng1767

[5] Rawlings AV, Harding CR. Moisturization and skin barrier function. Dermatol Ther. 2004;17 Suppl 1:43–8. 14728698 10.1111/j.1396-0296.2004.04s1005.x

[6] Smith FJD, Irvine AD, Terron-Kwiatkowski A, Sandilands A, Campbell LE, Zhao Y, et al Loss-of-function mutations in the gene encoding filaggrin cause ichthyosis vulgaris. Nat Genet. 2006;38(3):337–42. doi: 10.1038/ng1743 16444271 10.1038/ng1743

[7] Kezic S, Kemperman PMJH, Koster ES, de Jongh CM, Thio HB, Campbell LE, et al Loss-of-function mutations in the filaggrin gene lead to reduced level of natural moisturizing factor in the stratum corneum. J Invest Dermatol. 2008;128(8):2117–9. doi: 10.1038/jid.2008.29 18305568 10.1038/jid.2008.29

[8] Flohr C, England K, Radulovic S, McLean WHI, Campbel LE, Barker J, et al Filaggrin loss-of-function mutations are associated with early-onset eczema, eczema severity and transepidermal water loss at 3 months of age. Br J Dermatol. 2010;163(6):1333–6. 21137118 10.1111/j.1365-2133.2010.10068.x

[9] Weidinger S, O'Sullivan M, Illig T, Baurecht H, Depner M, Rodriguez E, et al Filaggrin mutations, atopic eczema, hay fever, and asthma in children. Journal of Allergy and Clinical Immunology. 2008;121(5):1203–9.e1. http://dx.doi.org/10.1016/j.jaci.2008.02.014. doi: 10.1016/j.jaci.2008.02.014 18396323 10.1016/j.jaci.2008.02.014

[10] Venkataraman D, Soto-Ramírez N, Kurukulaaratchy RJ, Holloway JW, Karmaus W, Ewart SL, et al Filaggrin loss-of-function mutations are associated with food allergy in childhood and adolescence. Journal of Allergy and Clinical Immunology. 2014;134(4):876–82.e4. doi: 10.1016/j.jaci.2014.07.033 25174864 10.1016/j.jaci.2014.07.033 PMC4186905

[11] van den Oord RAHM, Sheikh A. Filaggrin gene defects and risk of developing allergic sensitisation and allergic disorders: systematic review and meta-analysis. BMJ. 2009;339 doi: 10.1136/bmj.b2433 19589816 10.1136/bmj.b2433 PMC2714678

[12] Margolis DJ, Mitra N, Kim B, Gupta J, Hoffstad OJ, Papadopoulos M, et al Association of HLA-DRB1 genetic variants with the persistence of atopic dermatitis. Human immunology. 2015;76(8):571–7. Epub 2015/08/27. doi: 10.1016/j.humimm.2015.08.003 ; PubMed Central PMCID: PMCPMC4593755. 26307177 10.1016/j.humimm.2015.08.003 PMC4593755

[13] Liang Y, Chang C, Lu Q. The Genetics and Epigenetics of Atopic Dermatitis-Filaggrin and Other Polymorphisms. Clinical reviews in allergy & immunology. 2016;51(3):315–28. Epub 2015/09/20. doi: 10.1007/s12016-015-8508-5 . 26385242 10.1007/s12016-015-8508-5

[14] Kim SY, Yang SW, Kim HL, Kim SH, Kim SJ, Park SM, et al Association between P478S polymorphism of the filaggrin gene & atopic dermatitis. The Indian journal of medical research. 2013;138(6):922–7. Epub 2014/02/14. ; PubMed Central PMCID: PMCPMC3978983. 24521637 PMC3978983

[15] Wang IJ, Lin T-J. FLG P478S polymorphisms and environmental risk factors for the atopic march in Taiwanese children: a prospective cohort study. Annals of Allergy, Asthma & Immunology. 2015;114(1):52–7. doi: 10.1016/j.anai.2014.10.019 25528737 10.1016/j.anai.2014.10.019

[16] Sandilands A, Terron-Kwiatkowski A, Hull PR, O'Regan GM, Clayton TH, Watson RM, et al Comprehensive analysis of the gene encoding filaggrin uncovers prevalent and rare mutations in ichthyosis vulgaris and atopic eczema. Nat Genet. 2007;39(5):650–4. doi: 10.1038/ng2020 17417636 10.1038/ng2020

[17] Landrum MJ, Lee JM, Riley GR, Jang W, Rubinstein WS, Church DM, et al ClinVar: public archive of relationships among sequence variation and human phenotype. Nucleic Acids Res. 2014;42(Database issue):D980–5. doi: 10.1093/nar/gkt1113 24234437 10.1093/nar/gkt1113 PMC3965032

[18] Stenson PD, Mort M, Ball EV, Shaw K, Phillips A, Cooper DN. The Human Gene Mutation Database: building a comprehensive mutation repository for clinical and molecular genetics, diagnostic testing and personalized genomic medicine. Hum Genet. 2014;133(1):1–9. doi: 10.1007/s00439-013-1358-4 24077912 10.1007/s00439-013-1358-4 PMC3898141

[19] Xue Y, Chen Y, Ayub Q, Huang N, Ball EV, Mort M, et al Deleterious- and disease-allele prevalence in healthy individuals: insights from current predictions, mutation databases, and population-scale resequencing. Am J Hum Genet. 2012;91(6):1022–32. doi: 10.1016/j.ajhg.2012.10.015 23217326 10.1016/j.ajhg.2012.10.015 PMC3516590

[20] Adzhubei IA, Schmidt S, Peshkin L, Ramensky VE, Gerasimova A, Bork P, et al A method and server for predicting damaging missense mutations. Nat Methods. 2010;7(4):248–9. doi: 10.1038/nmeth0410-248 20354512 10.1038/nmeth0410-248 PMC2855889

[21] Czech W, Krutmann J, Schopf E, Kapp A. Serum eosinophil cationic protein (ECP) is a sensitive measure for disease activity in atopic dermatitis. Br J Dermatol. 1992;126(4):351–5. Epub 1992/04/01. . 1571256 10.1111/j.1365-2133.1992.tb00677.x

[22] Goto T, Morioka J, Inamura H, Yano M, Kodaira K, Igarashi Y, et al Urinary eosinophil-derived neurotoxin concentrations in patients with atopic dermatitis: a useful clinical marker for disease activity. Allergology international: official journal of the Japanese Society of Allergology. 2007;56(4):433–8. Epub 2007/10/30. doi: 10.2332/allergolint.O-07-489 . 17965582 10.2332/allergolint.O-07-489

[23] Severity scoring of atopic dermatitis: the SCORAD index. Consensus Report of the European Task Force on Atopic Dermatitis. Dermatology (Basel, Switzerland). 1993;186(1):23–31. Epub 1993/01/01. . 8435513 10.1159/000247298

[24] Landeck L, Visser M, Skudlik C, Brans R, Kezic S, John SM. Clinical course of occupational irritant contact dermatitis of the hands in relation to filaggrin genotype status and atopy. Br J Dermatol. 2012;167(6):1302–9. Epub 2012/09/12. doi: 10.1111/bjd.12035 . 22962861 10.1111/bjd.12035

[25] Ginger RS, Blachford S, Rowland J, Rowson M, Harding CR. Filaggrin repeat number polymorphism is associated with a dry skin phenotype. Arch Dermatol Res. 2005;297(6):235–41. doi: 10.1007/s00403-005-0590-8 16261374 10.1007/s00403-005-0590-8

[26] Meng L, Wang L, Tang H, Tang X, Jiang X, Zhao J, et al Filaggrin gene mutation c.3321delA is associated with various clinical features of atopic dermatitis in the Chinese Han population. PLoS ONE. 2014;9(5):e98235 doi: 10.1371/journal.pone.0098235 24858702 10.1371/journal.pone.0098235 PMC4032331

[27] Henderson J, Northstone K, Lee SP, Liao H, Zhao Y, Pembrey M, et al The burden of disease associated with filaggrin mutations: a population-based, longitudinal birth cohort study. J Allergy Clin Immunol. 2008;121(4):872–7.e9. Epub 2008/03/08. doi: 10.1016/j.jaci.2008.01.026 . 18325573 10.1016/j.jaci.2008.01.026

[28] Desmet FO, Hamroun D, Lalande M, Collod-Beroud G, Claustres M, Beroud C. Human Splicing Finder: an online bioinformatics tool to predict splicing signals. Nucleic Acids Res. 2009;37(9):e67 Epub 2009/04/03. doi: 10.1093/nar/gkp215 ; PubMed Central PMCID: PMCPMC2685110. 19339519 10.1093/nar/gkp215 PMC2685110

[29] Yeo G, Burge CB. Maximum entropy modeling of short sequence motifs with applications to RNA splicing signals. Journal of computational biology: a journal of computational molecular cell biology. 2004;11(2–3):377–94. Epub 2004/08/03. doi: 10.1089/1066527041410418 . 15285897 10.1089/1066527041410418

[30] Stein Gold LF, Eichenfield LF. Atopic dermatitis progression: evaluating intervention strategies. Seminars in cutaneous medicine and surgery. 2017;36(2 Suppl 2):S39–s41. Epub 2017/06/28. doi: 10.12788/j.sder.2017.010 . 28654709 10.12788/j.sder.2017.010

[31] Thijs JL, van Seggelen W, Bruijnzeel-Koomen C, de Bruin-Weller M, Hijnen D. New Developments in Biomarkers for Atopic Dermatitis. Journal of clinical medicine. 2015;4(3):479–87. Epub 2015/08/05. doi: 10.3390/jcm4030479 ; PubMed Central PMCID: PMCPMC4470140. 26239250 10.3390/jcm4030479 PMC4470140

[32] Murat-Susic S, Lipozencic J, Zizic V, Husar K, Marinovic B. Serum eosinophil cationic protein in children with atopic dermatitis. International journal of dermatology. 2006;45(10):1156–60. Epub 2006/10/17. doi: 10.1111/j.1365-4632.2006.02865.x . 17040428 10.1111/j.1365-4632.2006.02865.x

[33] Taniuchi S, Chihara J, Kojima T, Yamamoto A, Sasai M, Kobayashi Y. Serum eosinophil derived neurotoxin may reflect more strongly disease severity in childhood atopic dermatitis than eosinophil cationic protein. Journal of dermatological science. 2001;26(1):79–82. Epub 2001/04/27. . 11323224 10.1016/s0923-1811(00)00151-1

[34] Tomassini M, Magrini L, De Petrillo G, Adriani E, Bonini S, Balsano F, et al Serum levels of eosinophil cationic protein in allergic diseases and natural allergen exposure. J Allergy Clin Immunol. 1996;97(6):1350–5. Epub 1996/06/01. . 8648032 10.1016/s0091-6749(96)70204-x

[35] Wang IJ, Lin TJ, Kuo CF, Lin SL, Lee YL, Chen PC. Filaggrin polymorphism P478S, IgE level, and atopic phenotypes. Br J Dermatol. 2011;164(4):791–6. Epub 2011/01/12. doi: 10.1111/j.1365-2133.2011.10212.x . 21219289 10.1111/j.1365-2133.2011.10212.x

[36] Sullivan M, Silverberg NB. Current and emerging concepts in atopic dermatitis pathogenesis. Clinics in dermatology. 2017;35(4):349–53. Epub 2017/07/16. doi: 10.1016/j.clindermatol.2017.03.006 . 28709564 10.1016/j.clindermatol.2017.03.006

